# Buckwheat Cakes

**Published:** 1876-12

**Authors:** 


### BUCKWHEAT CAKES

And molasses, make a favorite winter dish for multitudes in winter time. Why not in summer also? We need in winter the food which contains most carbon; that is, the heat producing principle, something which will keep up the internal fires to compensate for the external cold. Meats, everything containing fat, are largely made of carbon, hence we instinctively eat heartily of meats iu winter, but have small appetite for them in summer. The same instinct receives greedily the buckwheat cakes in winter, and turns from them in summer, while other forms of bread materials, meal and flour, are desired all the year. It is because buckwheat cakes are superior to bread as to fatty matter, while the syrup and butter used with them are almost entirely of carbon. So that there is nothing more suitable for a winter morning's breakfast than buckwheat cakes and molasses. In New York, where almost every kitchen is under the same roof with the dining room and parlors, the fumes arising from the baking of the cakes on the ordinary iron instrument which requires greasing, are not very desirable; this may be obviated by using a soap-stone griddle, which does not require to be greased to prevent the cakes from sticking. Children and delicate persons should use the finest white flour of buckwheat. The robust, who exercise or work a great deal in the open air, should use the buckwheat flour which contains all the bran, because the bran is the richest part, yielding more nutriment and strength.

If any unfortunate dyspeptic cannot tolerate them, such an one has only to let them alone, and there will be more of this luxury left to those who can eat them with pleasure and impunity, having had the wit to avoid eating them like a glutton. The simple fact that any given item of food "is not good for" one man, does not "set well" on the stomach, is no proof that it is not positively benificial to others, it is simply a proof that it is not good for him. This is a practical thought of considerable importance.

